# Synergistic magnetoelectric enhancement in 0–3 particulate multiferroic composites: unveiling the exceptional interplay of Ba_0.85_Sm_0.15_TiO_3_ and Co_0.85_Sm_0.15_Fe_2_O_4_ phases for superior energy conversion†

**DOI:** 10.1039/d4ra01360c

**Published:** 2024-05-17

**Authors:** Showket Ahmad Bhat, Mohd Ikram

**Affiliations:** a Solid State Research Laboratory, Dept. of Physics, NIT Srinagar J&K 190006 India showketbht7@gmail.com

## Abstract

This study investigates the complex investigation of the 0–3 particulate multiferroic properties found in composite materials. The materials used in this study are (1 − *x*)Ba_0.85_Sm_0.15_TiO_3_ (SmBT)–*x*Co_0.85_Sm_0.15_Fe_2_O_4_ (SmCF), and the research utilizes a combination of the solid-state reaction method and mechanical milling techniques. A cubic spinel secondary phase was discovered in Co_0.85_Sm_0.15_Fe_2_O_4_, while a tetragonal structure was found in Ba_0.85_Sm_0.15_TiO_3_. The *M*–*H* loops clearly indicate the presence of exchange interactions between spins with varying orientations within the domains. As the proportion of SmCF in the composites increased, the saturation magnetization and magnetic moments became stronger, indicating a greater level of exchange interactions. Through a temperature-dependent analysis, an increase was observed in both the dielectric constant (*ε*_r_) and dielectric loss (tan *δ*). Nevertheless, there was a decrease in *ε*_r_ above the Curie temperature. It is worth mentioning that there was a significant increase in the magnetoelectric coupling constant, suggesting a stronger interaction between magnetic and electric fields. This increased interaction leads to a more effective conversion of electrical energy into its magnetic equivalent, indicating a significant improvement in overall energy efficiency. The results of our study shed light on a promising direction for the development of advanced multifunctional materials, which could have significant implications for energy-efficient applications.

## Abstract 2

This study investigates the complex investigation of the 0–3 particulate multiferroic properties found in composite materials.

## Graphical abstract

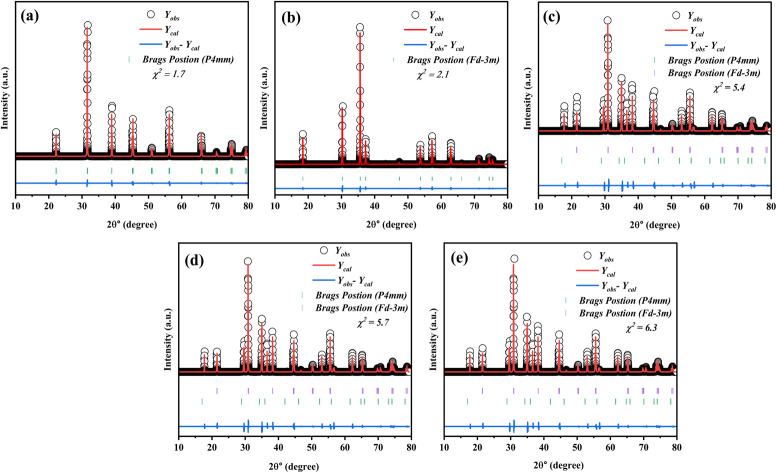

## Introduction

It is widely acknowledged that one of the key forces influencing development and economic progress is the advancement in materials sciences. Research and advancements in material science have had a profound impact on our society since the time it led to the industrial revolution in silicon in the 1950s.^1^ A substance acquires multifunctionality in materials science when it has many useful properties. Multiferroics are a class of materials that possess multiple fundamental ferroic states, such as ferroelasticity, ferroelectricity, ferromagnetism, and ferrotoroidicity. The multiferroic materials are those that demonstrate the presence of at least ferroelectric and ferro/ferrimagnetic orders, these ferroic orders have attracted much attention from the scientific community.^2^ Ferroic materials tend to change their internal alignment in response to an external field. External electrical and magnetic forces have the ability to influence the alignment of electrical dipoles and spins in materials that can be polarized. A variety of connection strategies, including 0–3 particulate, 2–2 laminated, and 1–3 fiber rod composites, were utilised to regularly produce artificial multiferroic composites with unique functional characteristics.^3^ Among these, fabricating the 0–3 particle composite is very simple as it requires no complex equipment. So far, numerous 0–3 particle composites have been reported, and different ferroic and magneto-dielectric characteristics have been covered in-depth.^4–7^

Among the different methods, the 0–3 particulate composite method is often used to develop multiferroic composites with better ferroic characteristics. By carefully selecting a ferroelectric phase with low leakage currents^8^ and a magnetic phase with a high magnetic moment and magnetostriction, it is possible to achieve maximum strain transfer from the magnetic to electric phase. This leads to an enhanced magnetoelectric response across the boundary interface. magnetic moment and magnetostriction.^9^ This strategy could result in better ME performance for the composite material. The ratio of the polarization (*P*) caused by the applied magnetic field is the measure of the multiferroic composites' ME response (*H*).

The primary parameter utilized in assessing experimental data and developing different multiferroics-based applications is the voltage magneto-electric coefficient (*α*). The voltage magneto-electric coupling coefficient and the magnetically induced coupling coefficient have the following relationship: *αH* = *ε*_o_*ε*_r_*α*_H_^*V*^. In SI units, [S m^−1^] is used to denote both *α*_H_ and *α*_E_. However, the more useful voltage magnetoelectric coefficient, *α*_H_^*V*^ (see relation (4)), is represented as [*V*/A] in SI units and as [*V*/cm Oe] in CGS units, which are likewise used in the majority of practical applications and scientific measurements.^10^

Composites made from perovskite oxides like PbTiO_3_ and BaTiO_3_ demonstrate notable enhancements in various properties, encompassing dielectric constant, impedance, energy storage, and *M*–*E* coupling, through the utilisation of ionic location, deformation, and orientation. Although PbTiO_3_ composites possess impressive dielectric and piezoelectric properties, their environmental impact is concerning due to their toxic nature. Thus, for the protection of the environment, it is critical to investigate lead-free ceramics.^11^ Thus, multiphase multiferroic composites were manufactured artificially and were found to exhibit a strong ME effect. When it comes to technology, these materials with a strong ME effect were found to provide better results than single-phase materials.^12^ The activation of the ME effect in composite materials occurs through the transfer of mechanical strain at the interfaces between the two ferroic phases. In addition, the ME composite preserves the unique characteristics of each ferroic phase.^4^

The materials Ba(Ti, Sn)O_3_–(Ba, Ca)TiO_3_ (with a piezoelectric coefficient *d*_33_ ∼ 550 pC N^−1^)^13^ and Ba(Ti, Hf)O_3_–(Ba, Ca)TiO_3_ (with a piezoelectric coefficient *d*_33_ ∼ 550 pC N^−1^)^14^ have been studied. The elevated piezoelectric response can be attributed to the 0.5BZT–0.5BCT or BCTZ composition, which aligns with the morphotropic phase boundary (MPB) composition. This results in a decrease in free energy anisotropy, similar to what is observed in PZT. Ba_0.85_Ca_0.15_Ti_0.9_–Zr_0.1_O_3_, also known as 0.5BZT–0.5BCT, has been identified as a promising substitute for traditional piezoelectric materials that contain lead. Ferroelectric materials based on ABO_3_ with a significant piezoelectric coefficient and ferrites exhibiting high magnetostriction are preferred for the purpose of achieving a robust magnetoelectric coupling effect. Researchers have extensively documented the piezoelectric effect in BCT-based ABO_3_. This material shows great potential for the development of lead-free multiferroic composites and has the ability to produce a significant ME response. BCT exhibits a Curie temperature of approximately 263 ± 10 K, which is lower than that of BaTiO_3_, which has a Curie temperature of around 293 K.^13,15–17^ At room temperature, the ferroelectric crystal structure undergoes a transition in symmetry from rhombohedral to orthorhombic and subsequently to tetragonal, with an increase in BCT content.^16^ Similarly, cobalt ferrite (CoFe_2_O_4_) is known to possess exceptional magnetic properties, with the highest magnetostriction coefficient (*k* ∼ −110 × 10^−6^) among all ferrite families. Additionally, it exhibits a high Curie temperature. Hence, the investigation of a composite structure comprising BCT and CoFe_2_O_4_ (CFO) is a promising avenue to pursue. Sadhana *et al.* have produced nanocomposites of *x*Ba_0.8_Ca_0.2_TiO_3_–(1 − *x*)Ni_0.2_Cu_0.3_Zn_0.5_Fe_2_O_4_, where *x* values of 0.1, 0.3, 0.5, 0.7, and 0.9 were used. Their findings indicate that the highest magnetoelectric coefficient of 280 mV cm^−1^ Oe^−1^ was observed at a composition of *x* = 0.3.^18^ Leonel *et al.*, conducted a study on the structural characterizations of composite materials consisting of barium titanate–cobalt ferrite (BaTiO_3_–CoFe_2_O_4_). Their findings revealed a correlation between vacancies, interfacial stress, and the reduction of tetragonal distortion in the ferroelectric structure.^19^ BCZT85–CFO15 have potential contenders for the development of lead-free materials that exhibit increased ME coupling at ambient temperature. The ME coupling coefficient was found to be approximately 6.03 ps m^−1^.^20^ The solid-state reaction method to synthesize (1 − *x*)BaTiO_3_–(*x*)CoFe_1.8_Zn_0.2_O_4_, where *x* = 10, 20, 30, and 40 weight percentages, found that an increase in ferrite fraction results in an increase in both morphology and dielectric constant.^21^ A wet chemical method was used to produce a series of materials with varying concentrations of (1 − *x*)(Ba_0.8_Ca_0.2_TiO_3_)–*x*(Co_0.6_Zn_0.4_Fe_2_O_4_) (where *x* = 0.00, 0.01, 0.02, 0.03, 0.04, and 1.00) was found that the measured leakage current values conform to the ohmic conduction mechanism across all samples. Additionally, as the concentration of ferrite increased, the saturation magnetization (*M*_s_) also increased, reaching its maximum value at a concentration of *x* = 0.04.^22^

When samarium is introduced into a host material, it brings about significant changes in its electronic and magnetic properties, resulting in the emergence of new functionalities. Recent studies on samarium-doped materials have made significant progress in understanding their magnetization,^23^ ferroelectricity,^24^ and magnetostriction.^25^ The unique properties of samarium make it a highly desirable choice for a wide variety of uses, including magnetic data storage, sensors, and actuators. Furthermore, it is important to highlight that samarium demonstrates a significant level of abundance and cost-effectiveness, making it a highly attractive alternative to more expensive dopants like rare earth metals. The composite materials (1 − *x*)Ba_0.85_Sm_0.15_TiO_3_–*x*Co_0.85_Sm_0.15_Fe_2_O_3_ (*x* = 0.0, 0.02, 0.04, 0.06) were synthesized through the solid-state reaction method. The present study aimed to investigate the structural, temperature dependent dielectric, magnetic properties, and ME coupling of composite materials composed of (1 − *x*)Ba_0.85_Sm_0.15_TiO_3_–*x*Co_0.85_Sm_0.15_Fe_2_O_3_.

## Experimental details

In this study, we employed a combination of the solid-state reaction method and mechanical milling technique to fabricate samples of a multiferroic material composed of samarium-doped CoFe_2_O_4_ and BaTiO_3_. The high-grade chemicals from Sigma-Aldrich, including BaCO_3_, TiO_2_, Sm_2_O_3_, and acetone, were used as raw materials for the synthesis of the ferroelectric phase, specifically the samarium-doped barium titanate (Ba_0.85_Sm_0.15_TiO_3_). Materials were thoroughly mixed with the milling balls to ensure a uniform distribution of the components. The raw materials have been carefully arranged into three vials, each with a capacity of 20 grams, following a predetermined composition. The composition analysis of Ba_0.85_Sm_0.15_TiO_3_ was conducted using stoichiometric principles. The sample underwent mechanical activation through milling using a high-energy ball mill, specifically the Spex 8000D. The experimental protocol dictated a predetermined milling time of 5 hours to mitigate the potential contamination of the sample arising from the milling medium. In this experimental study, the ball-to-powder weight ratio was carefully set at 10 : 1. Afterwards, the mixtures underwent a drying process in an oven for a period of 5 hours. The experimental procedure involved a cyclical process consisting of 90 minutes of milling followed by a subsequent 30 minute period of standby. Ultimately, the mixtures underwent sintering at a temperature of 1000 °C for a duration of 5 hours with a heating rate of 5 °C min^−1^. A follow-up grinding step was performed to reduce the clumping of particles caused by the sintering process. The grinding process was conducted using a mortar pestle. The careful grinding procedure was performed to effectively disperse and isolate the particles, resulting in improved uniformity and excellence of the sintered material (Ba_0.85_Sm_0.15_TiO_3_). The mentioned powder is then analyzed extensively to meet the predetermined objectives.

A similar method was employed to analyze the magnetic phase of a specific material, namely samarium doped cobalt ferrite (Co_0.85_Sm_0.15_Fe_2_O_4_) with sintering temperature of 850 °C for a duration of 4 hours with a heating rate of 5 °C min^−1^. This material was synthesised using high-quality chemicals sourced from Sigma-Aldrich, including Co_3_O_4_ (99.9 percent), Fe_2_O_3_ (99.9 percent), and Sm_2_O_3_ (99.9 percent). The powder is thoroughly analyzed to meet the predetermined objectives.

In addition, the procedure for the particulate composite of (1 − *x*)Ba_0.85_Sm_0.15_TiO_3_–*x*Co_0.85_Sm_0.15_Fe_2_O_3_ (*x* = 0.0, 0.02, 0.04, 0.06) remained consistent. The synthesised powder was then converted into pellets using 13 mm and 8 mm dyes of the pelletizer machine. The pellets underwent a sintering process at a temperature of 1200 °C for a duration of 10 hours, with the specified heating rate, and were then allowed to cool naturally. The powder is thoroughly analyzed to meet the predetermined objectives.

The equations provided below represent the reaction stoichiometry for the given particulate multiferroic composites. The RE ions have the ability to replace both A and B sites in the perovskite lattice, depending on the Ba/Ti ratio and the ionic radius of the RE element. This phenomenon is observed due to the amphoteric nature of the dopants.^26^ Here is a description of the charge compensation processes involved in replacing the RE oxide (M_2_O_3_, where M is the RE metal) at the A and B sites of BaTiO_3_.^27^
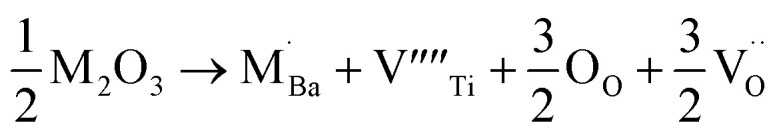

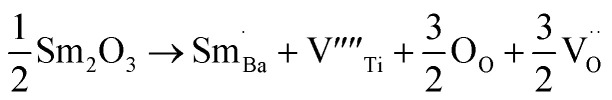

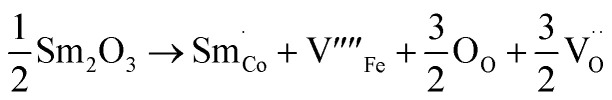

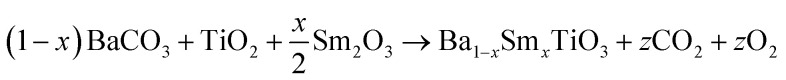

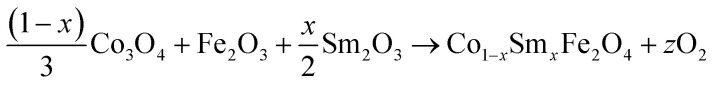
(1 − *X*)Ba_1−*x*_Sm_*x*_TiO_3_ + *X*Co_1−*x*_Sm_*x*_Fe_2_O_4_ → (1 − *X*)Ba_1−*x*_Sm_*x*_TiO_3_−*X*Co_1−*x*_Sm_*x*_Fe_2_O_4_where the *x* = 0.15 and *X* = 0.0, 0.02, 0.04, 0.06 and *z* is some constant which can take both natural as well as fraction values.

The phase confirmation and structural parameters of the synthesised composite materials were examined using a Rigaku X-ray diffractometer equipped with Cu Kα (*λ* = 1.5406 Å). Obtaining Raman spectra was made possible through the use of advanced scientific equipment, including a T64000 Horiba-Jobin Yvon triple-monochromator spectrometer with a confocal microscope and a liquid N_2_ cooled charge coupled device (CCD). By utilising an argon ion laser with a wavelength of 514.5 nm, the scattering phenomenon was induced. To avoid sample heating, the laser's spot size was set to 1 μm^2^ using a 100× objective lens, while maintaining a fixed power of 1.0 mW. Kratos Analytical Ltd, AXIS Supra, X-ray source with a single color: the energy level of Al Kα is 1486.6 eV. An achromatic X-ray source that combines the Al Kα and Mg Kα wavelengths was utilised for X-ray photoelectron spectroscopy. A 2.7 tesla Vibrating Sample Magnetometer (VSM) Model Micro-Sense (USA) was utilised to conduct the analysis of magnetic properties. We used the LCR meter (Agilent 4284 A) in conjunction with a temperature controlling boiler from Lake Shore to examine the dielectric behaviour and ferro-electric transitions. A dynamic approach was employed to investigate the interaction between the electric and magnetic phases using alternating current (AC) and direct current (DC) magnetic fields.

### X-ray diffraction

**Fig. 1:**
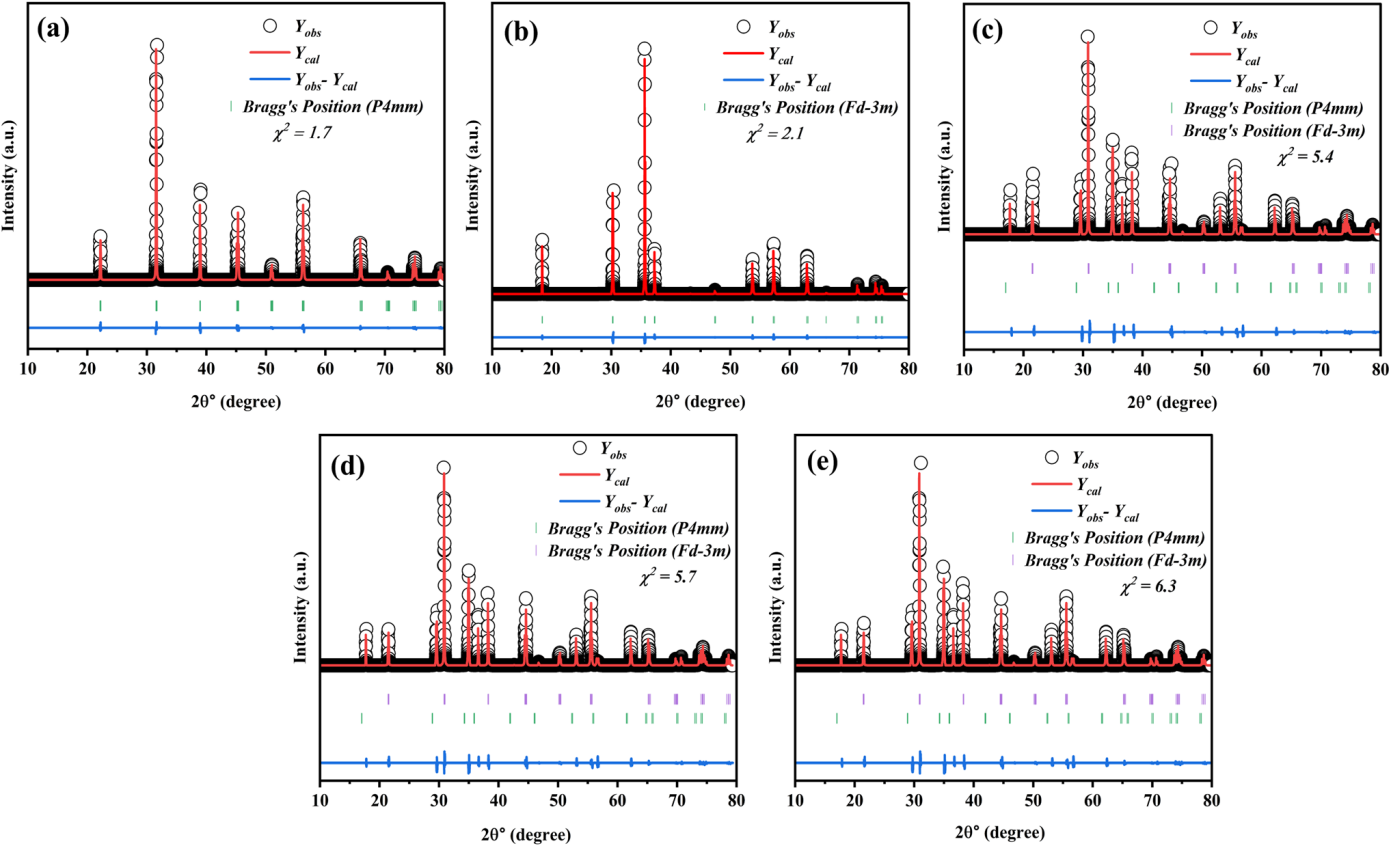
Rietveld refinement in the 2*θ* range of 10–80° of (1 − *x*)SmBT–*x*SmCF where (a) *x* = 0.00, (b) *x* = 1.00, (c) *x* = 0.02, (d) *x* = 0.04, and (e) *x* = 0.0.06 composites.

The XRD pattern in Fig. 1 illustrates the Rietveld refinement of the composite samples, highlighting the distinct growth of both phases across various compositions. Based on the XRD pattern, it is evident that the composites exhibited a polycrystalline structure. The XRD pattern shows that the composition (1 − *x*)SmBT–*x*SmCF (*x* = 0.0, 0.02, 0.04, 0.06) displays a mixture of tetragonal and cubic (spinel) structures. The XRD pattern peaks of SmBT and SmCF correspond to the JCPDS no. 811289 for BaTiO_3_ and the JCPDS no. 22-1086 for CoFe_2_O_4_ phase. From the observed patterns in the files, it can be inferred that the samples display the tetragonal phase at room temperature. The XRD patterns of all composites exhibit a combination of cubic spinel and tetragonal structures, indicating a lack of observable bias or deformation in the structure. After examining the XRD patterns, it was observed that all composites showed peaks that matched SmBT and SmCF. Through the analysis of the prominent peak in the individual samples of the composite materials, it becomes evident that both phases are present in the composite samples at the same time. As the weight percentage of one component increased in the other, there was a noticeable change in the intensity of the peaks. The peaks associated with the increasing component became more pronounced, while the peaks associated with the decreasing component became less prominent.

Additional analyses have been performed on the crystal structure of all the samples using Full-Prof software, employing the Rietveld refinement technique. The structure of the SmCF compound is spinal, displaying cubic symmetry and space group *Fd*3̄*m*. On the other hand, the SmBT compound showcases a perovskite structure with tetragonal symmetry and space group *P*4*mm*. The XRD patterns of all the samples were analyzed using a full-prof approach, which utilizes space groups and an approximate structural model of these structures. Table 1 provides a summary of refined lattice parameters, along with their corresponding residual factor *R*_p_, goodness of fit *χ*^2^, and weighted residual factor *R*_wp_. Given the similarity in ionic radii between Fe^3+^ and Co^2+^ in the B site of the ferrite phase, and Ti^4+^ in the B-site of the electric phase, it is possible that ion diffusion could occur during the sintering process. This could potentially explain the modification of lattice parameters in the ferroelectric–ferrite phase.

**Table 1:** Refined lattice parameters and residual factor (*R*_p_), goodness of fit (*χ*^2^), and weighted residual factor (*R*_wp_) of (1 − *x*)SmBT–*x*SmCF

Sample	*a* (Å)	*c* (Å)	*c*/*a*	*V* (Å)^3^	*D* (Å)	*R*_f_	*R*_p_	*R*_wp_	*χ* ^2^
SmCF	8.382 ± 0.015	8.382 ± 0.017	1	588.90 ± 0.013	446.4	1.69	5.13	12.6	1.7
SmBT	3.980 ± 0.0112	4.201 ± 0.0152	1.056	66.64 ± 0.0112	309.8	1.81	5.13	13.2	2.1
SmBT–SmCF (0.02)	3.852 ± 0.0129	4.254 ± 0.0157	1.105	63.12 ± 0.0119	397.2	2.82	5.13	12.6	5.4
SmBT–SmCF (0.04)	3.850 ± 0.0135	4.257 ± 0.0129	1.106	63.09 ± 0.0122	428.5	2.02	5.13	12.6	5.7
SmBT–SmCF (0.06)	3.846 ± 0.0182	4.260 ± 0.0119	1.107	63.012 ± 0.0131	471.4	2.01	4.88	9.87	6.3

### FESEM and EDS

**Fig. 2:**
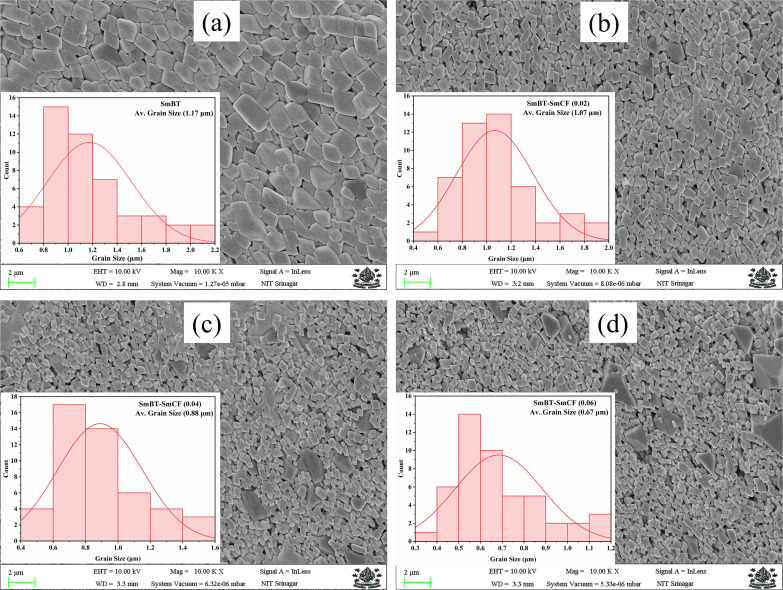
FESEM images and inset histograms for average particle size of (1 − *x*)SmBT–*x*SmCF (a) *x* = 0.0, (b) *x* = 0.02, (c) *x* = 0.04, (d) *x* = 0.06.

**Fig. 3:**
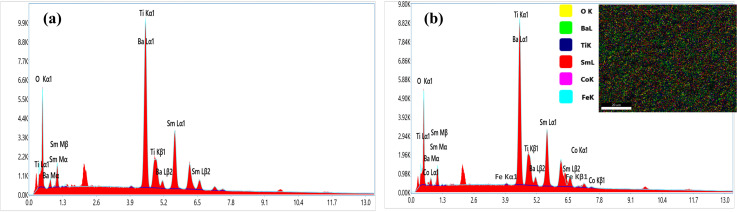
EDS spectrograms of (1 − *x*)SmBT–*x*SmCF (a) *x* = 0.0, (b) *x* = 0.06.

In order to elucidate the morphology and approximate particle size of the synthesised magnetic (1 − *x*)SmBT–*x*SmCF (*x* = 0.0, 0.02, 0.04, 0.06), a field emission scanning electron microscope (FESEM) images were acquired. The analysis conducted using ImageJ software revealed the presence of an inhomogeneous morphology in the product, as depicted in Fig. 2(a)–(d). The particles exhibited an uneven diameter distribution within the range of 1.17–0.67 μm, as depicted in the insets of Fig. 2 as a histogram with a normal distribution curve. The EDS spectrograms in Fig. 3(a) and (b) show the presence of all elements in the composites, including the (1 − *x*)SmBT–*x*SmCF phases. Based on the observation, it can be inferred that the desired elemental composition was successfully achieved during the synthesis process. In addition, the EDS analysis revealed that there were no impurities added to the composites while they were being sintered.

### Raman spectroscopy

**Fig. 4:**
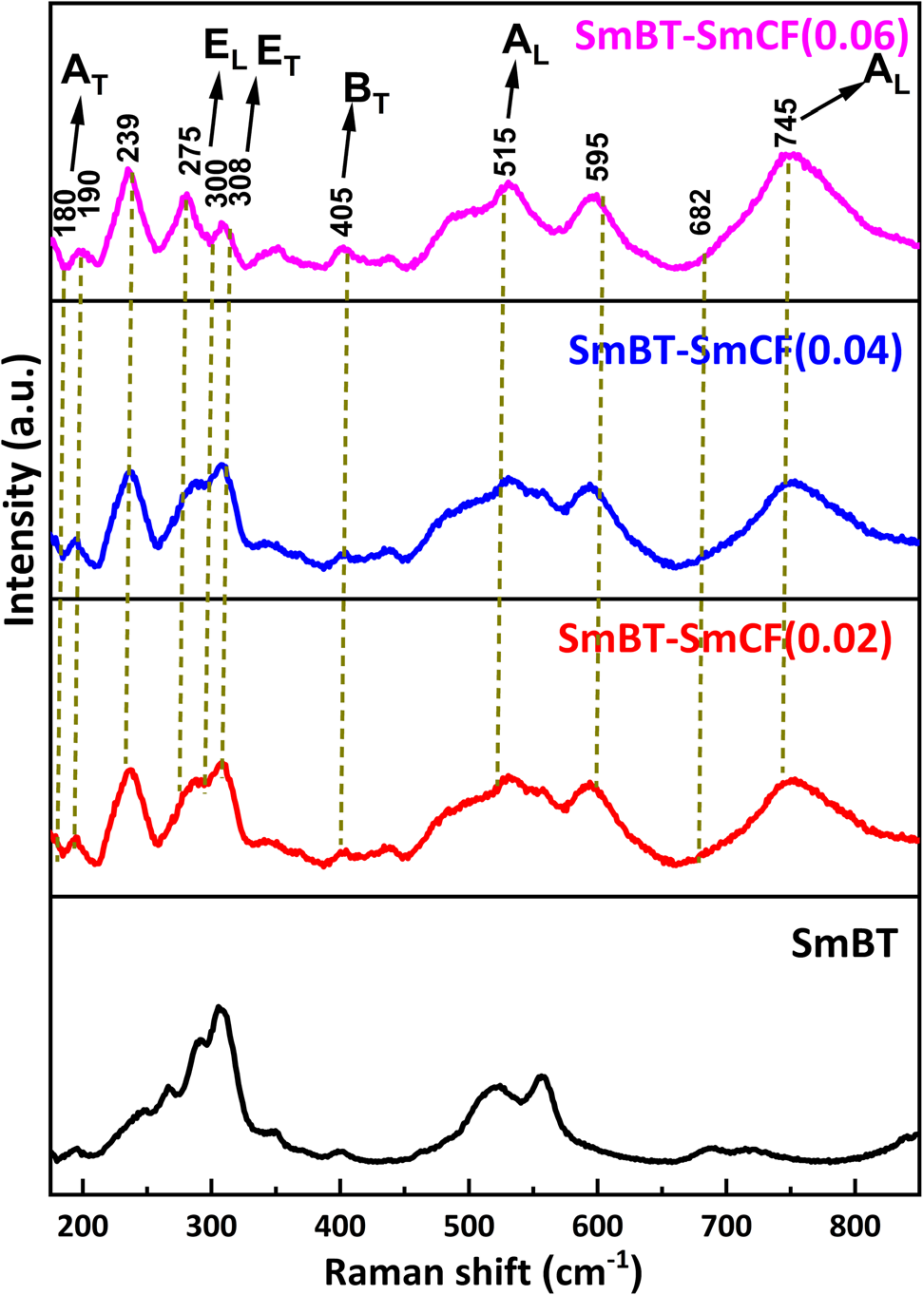
Raman spectra of composites (1 − *x*)SmBT–*x*SmCF (*x* = 0.0, 0.02, 0.04, 0.06).

Raman spectra are an incredibly sensitive spectroscopic technique that allows for the examination of the local structure of atoms and materials. Fig. 4 represents the Raman spectra of the SmBT and its composites from 150 to 800 cm^−1^. In a cubic perovskite structure, optical-vibration is triply degenerate in F_1u_ and F_2u_ modes. As there are fewer asymmetries in the cubic phase, F_1u_ mode is Raman inactive. Whereas by group theory F_1u_ and F_2u_ modes splits into A, E and B, E modes respectively in tetragonal phase as 4E + 3A_1_ + B_1_.^28^ The analysis of group theory reveals that the F_1u_ normal mode in the tetragonal phase undergoes a splitting into two modes, namely the A_1_ (non-degenerate) and E (doubly degenerate) types in the tetragonal phase. The F_2u_ type exhibits a separation of its mode into two distinct categories, namely the B type, which is non-degenerate, and the E type. It is a well-established fact that infrared activity is exhibited by the normal modes of type A and E, whereas type B does not display any infrared activity. Conversely, all the modes of A_1_, B_1_, and E is Raman active. Hence, it is possible to observe 8 Raman lines and 7 infrared absorption bands in tetragonal Raman spectrum of BaTiO_3_ crystals.^29^ The long range of electrostatic forces acting on these modes as they exist in transverse and longitudinal modes.^11^

The Raman spectra of SmBT–SmCF (0.02), SmBT–SmCF (0.04) and SmBT–SmCF (0.06) show optical modes are visible around at 190, 239, 308, 721 cm^−1^ which validate the symmetry of the tetragonal *P*4*mm* crystal.^30^ The metal–oxygen vibration of phonons and transverse non-symmetry vibration (A_T_) are found to be correlated at a frequency of 180 cm^−1^. The inclusion of SmCF in SmBT leads to lattice disorder, resulting in a spectrum observed between 200 and 275 cm^−1^.^31^ E_L_ and E_T_ modes are present at 306 cm^−1^ and at 745 cm^−1^ symmetric stretching A_L_ modes are present that shows resemblance to the tetragonal ferroelectric phase of BaTiO_3_. At 515 cm^−1^ a transverse A_L_ mode of TiO_6_ present. The peak's existence at 682 cm^−1^ ensures the composite's validity. The metal–oxygen migration at the tetrahedral sites in CoFe_2_O_4_ is shown by the origin of the frequency peak at 678 cm^−1^.^32^ The process of analysing Raman spectra included deconvoluting the acquired spectra by utilizing Lorentzian line-shape functions. The objective was to precisely align the Raman spectral peaks by means of a least-squares fitting algorithm. The Lorentzian fits are presented in ESI,† with particular emphasis on the scenario where the concentration of cobalt ferrite is *x* = 0.00, 0.06. The wavenumber corresponding to each vibrational mode was determined by utilizing the peak positions of the fitted Lorentzian.^33^

### X-ray photoelectron spectroscopy

**Fig. 5:**
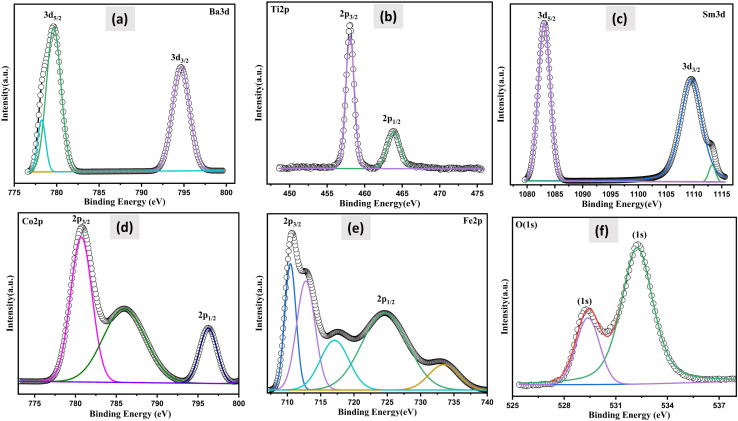
The deconvoluted XPS spectra of: (a) Ba 3d (b) Ti 2p (c) Sm 3d (d) Co 2p (e) Fe 2p and (f) O 1s measured on the (1 − *x*)SmBT–*x*SmCF core–shell powders.

XPS investigations were carried out, to ascertain the valence of the cations and their chemical makeup in order to assess the effect of SmCF on the magnetic characteristics of the (1 − *x*)SmBT–*x*SmCF multiferroic composites. The sample's thorough XPS survey spectra under investigation are presented in ESI.† The spin–orbit interaction induces a splitting of the Ti 2p peak into 2p_3/2_ and 2p_1/2_ components and rest of the elements. The difference between these two peaks is commonly used to quantify the magnitude of the spin–orbit splitting for Ti and other elements. Fig. 5 displays the estimated value of the spin–orbit splitting, as determined from the measured difference between the Ti 2p_3/2_ and Ti 2p_1/2_ peaks. All the predicted elements have been identified by analyzing the spectra, which includes barium (Ba), samarium (Sm), iron (Fe), titanium (Ti), cobalt (Co), and oxygen (O). By deconvoluting the core level spectra of the (1 − *x*)SmBT–*x*SmCF multiferroic composites (Fe 2p, Co 2p, Ti 2p, Ba 3d, O 1s and Sm 3d), Voigt functions (Lorentzian and Gaussian widths) with various inelastic backgrounds for each component were used.^34,35^ The XPS data analysis involved calibrating the binding energies of all the peaks with respect to the C 1s binding energy, which was determined to be 284.8 eV.

Two doublets were used to match the Fe 2p main peak, with binding energies of 710.5 eV and 733.4 eV, respectively, assigned to Fe^3+^ ions in octahedral and tetrahedral locations.^36^ Fe^2+^ ions were estimated to be at 724.6 eV. Fe 2p_3/2_ has a satellite peak that is distinct that is around 14 eV higher than the main peak. The Co 2p XPS spectra, which had binding energies of 779.7 eV and 796.4 eV and were assigned to Co^2+^ ions in octahedral sites and tetrahedral sites, respectively, were similarly fitted. The satellite peak of the Co 2p_3/2_ main line was also ascribed to the signal at 785.9 eV. At around 464 eV, there is a notable broad bump that is the peak of Ti 2p_1/2_. The peaks at 457.9 and 463.71 eV, which correspond to Ti^4+^, are attributed to Ti 2p_3/2_ and Ti 2p_1/2_, respectively.^37^ The Ba 3d level consists of two peaks, Ba 3d_3/2_ and Ba 3d_5/2_, with binding energies of 794.81 and 779.45 eV, respectively, and a peak difference of 15.36 eV, indicating that both barium atoms are in the Ba^2+^ state.^38^ It has been observed that the oxygen-deficient area is associated to the peak for O 1s spectra between 529.38 and 532.24 eV. The primary component at 532.24 eV is shown by the fitted and deconvoluted oxygen spectra, which indicates the decrease of oxygen-deficient species.^39^ Also, the de-convolution analysis performed on the synthesized sample, Sm^3+^ was found to be present in the 3d_5/2_ and 3d_3/2_ states with binding energies of 1083.08 eV and 1109.43 eV, respectively. Furthermore, Sm^2+^ (3d_3/2_) was also detected in the sample, exhibiting a binding energy of 1113.08 eV. The presence of both Fe^2+^ and Fe^3+^, and Sm^2+^ and Sm^3+^ within a sample is a result of the oxygen vacancies in the material influencing the oxidation state of the elements. Furthermore, the electrical conduction/transportation process in the material can be affected by the presence of oxygen vacancies, as they can create pathways for the movement of charged species, such as electrons or ions, which can influence the material's electrical properties.^40^ The atomic composition of all the samples under examination was established by adjusting the integral areas acquired *via* the deconvolution procedure to the XPS atomic sensitivity parameters listed in table presented in ESI.†^41^ The comprehensive XPS analysis, along with the identified valence states, offers a complete grasp of the changing chemical environment and oxidation states as the saturation of composites increases. This helps to reveal the complex connection between composition, valence, and magnetic properties in the (1 − *x*)SmBT–*x*SmCF multiferroic composites.

### Magnetic studies

**Fig. 6:**
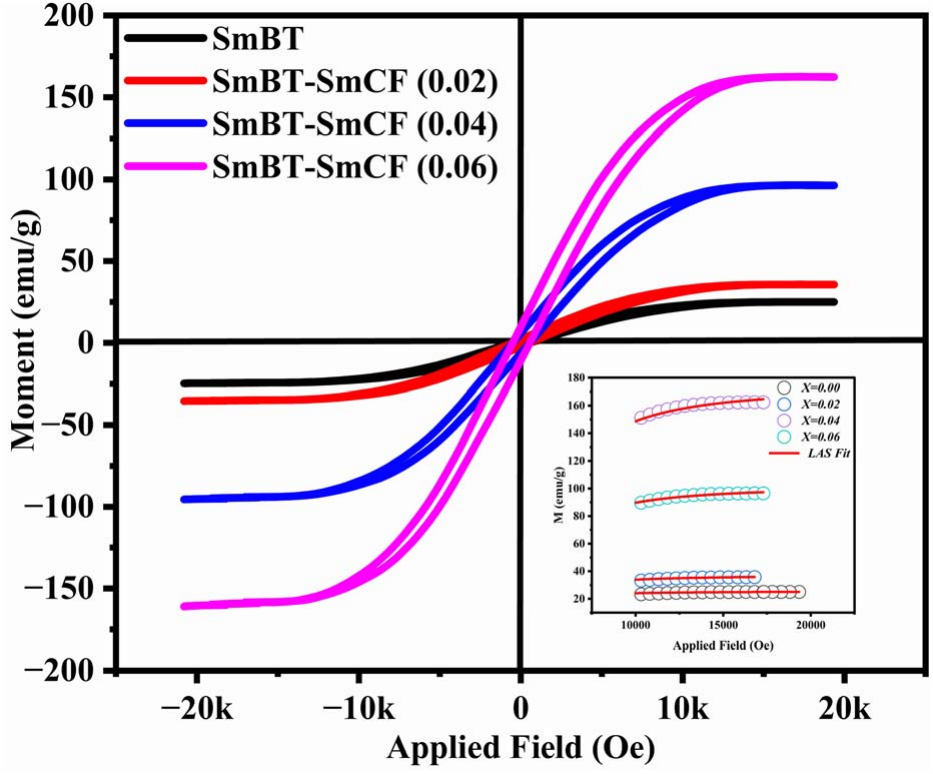
*M*–*H* loops and inset shows the LAS fit of particulate multiferroic composite of (1 − *x*)SmBT–*x*SmCF (*x* = 0.0, 0.02, 0.04, 0.06).

**Fig. 7:**
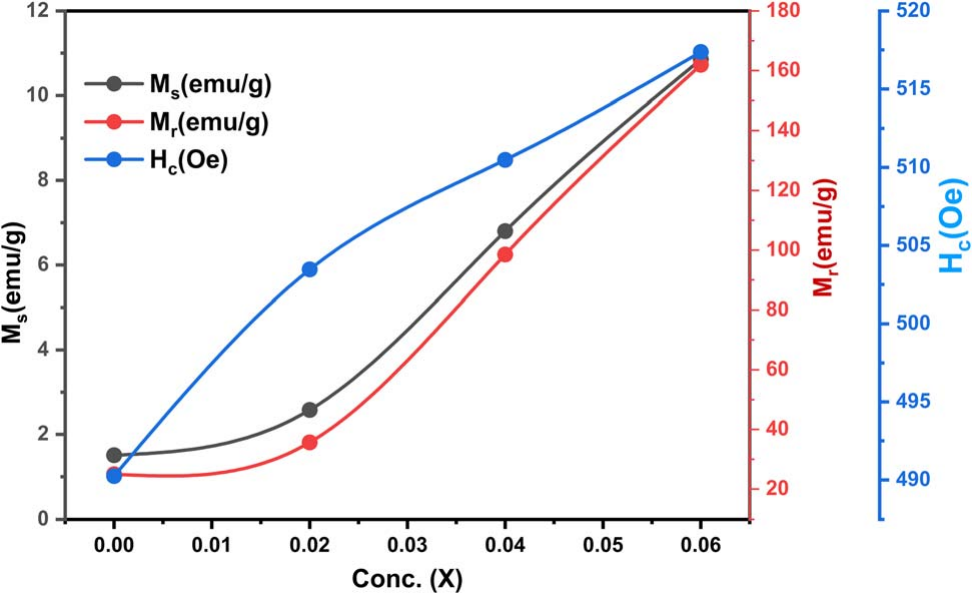
*M*_s_, *M*_r_ and *H*_c_ as a function of ferrite concentration (SmCF) in ferroelectric (SmBT).

The ferrimagnetic behavior of the SmBT phase and its particulate composites [(1 − *x*)SmBT–*x*SmCF (0.02, 0.04, 0.06)] at room temperature was confirmed by the saturated *M*–*H* loops as shown in Fig. 6 and the corresponding parameters are displayed in Table 2 (1 − *x*)Ba_0.85_Sm_0.15_TiO_3_–*x*Co_0.85_Sm_0.15_Fe_2_O_3_. All of the SmBT–SmCF composites and the pure SmBT phase shows the typical hysteresis loops, indicating that there is exchange interaction between spins with different orientations within domains. As the proportion of the magnetic SmCF ferromagnetic phase increases in the composites, the exchange interactions between different spins become stronger, resulting in an increase in saturation magnetization (*M*_s_) and magnetic moments (*μ*)^42^ shown by the Fig. 7. This trend is in accordance with the mass ratios of the SmBT phase used in the synthesis. The remnant magnetization (*M*_r_) in the composites also exhibits this same behavior, thereby provides reassurance that the ferroelectric SmBT phase causes limitations to prevent the bulk numbers of spins from being orientated in the field direction. One potential explanation for the increased magnetoelectric response is that this causes strain inside the composites.^43^

**Table 2:** Various parameters of magnetic studies of particulate multiferroic composite of (1 − *x*)SmBT–*x*SmCF (*x* = 0, 0.02, 0.04, 0.06)

Conc. *X*	*M*_s_ (emu g^−1^)	*M*_r_ (emu g^−1^)	*M*_r_/*M*_s_	*H*_c_ (Oe)	*K* (J m^−3^) ×10^4^	*μ*_exp_ (*μ*_B_)
*X* = 0	25.03	1.51	0.060	490.25	0.256	1.07
*X* = 0.02	35.67	2.58	0.072	503.47	0.394	1.53
*X* = 0.04	98.47	6.80	0.069	510.48	1.129	4.25
*X* = 0.06	161.97	10.85	0.067	517.37	2.190	7.02

The study of domain structures of the SmBT phase and its composites were analyzed by calculating the square–ness ratios (*M*_r_/*M*_s_). The results showed that all the samples of SmBT as well as its composites have multi-domain structure as the value of (*M*_r_/*M*_s_ < 0.5).^44^ The small values of *M*_r_/*M*_s_ in the samples may be attributed to the magnetic domains pinning effect, which is caused by the SmBT ferroelectric phase by blocking the spins from orienting in the field direction. In order to compute the magneto-crystalline anisotropy for such confined magnetic multi-domains, we used the Law of Approach to Saturation (LAS)^45^ in eqn (1) as shown in Fig. 6 inset.1
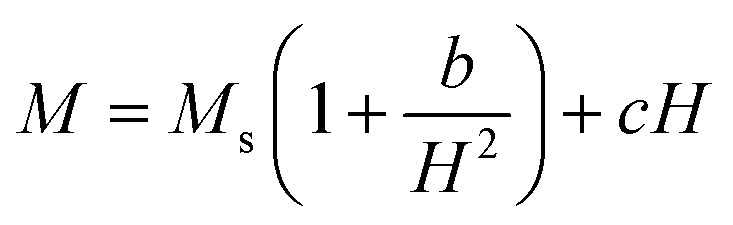


The *b*/*H*^2^ term in the equation is related to the magneto-crystalline anisotropy, which is a measure of the energy difference between different orientations of a magnetic moment within a crystal lattice. This term is dependent on the crystal structure and the atomic magnetic moments within the lattice. The *cH* term is referred to as a paramagnetic-like term and is associated with the high field magnetization. This term is related to the susceptibility of the material to an applied magnetic field and the alignment of the atomic magnetic moments within the material are written in eqn (2).2
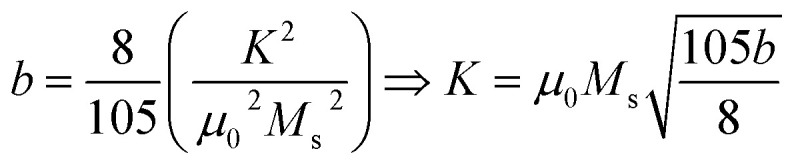
Here (eqn (6)) *μ*_o_ (permeability of free space) = 4π × 10^−7^ H m^−1^, *K* is the magneto-crystalline anisotropy constant which is measure of the energy difference between the easy axis and the hard axis of a ferromagnetic material. The magneto-crystalline anisotropy constant (*K*) increases with the increase in ferrite concentration in Sm-based ternary (SmBT) composites. This is due to the increase in Co^2+^ ions in the composites, which results in stronger exchange interactions between Co^2+^ and Fe^3+^ ions.^2,46^ These interactions lead to an increase in the alignment of the magnetic moments, leading to an increase in *K*.^47^ The magnetic moment (*μ*_exp_)^48^ experimentally is also calculated by the expression (3).3
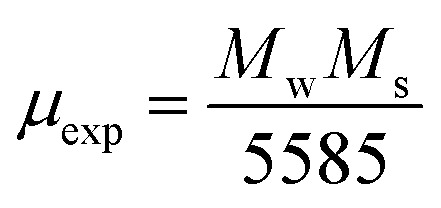
where *M*_w_ is molecular weight of samples.

### Dielectric studies

The dielectric constant (*ε*_r_) and dielectric loss (tan *δ*) of the sintered pellets of thickness ‘*d*’ and area ‘*A*’ were evaluated relations (4) and (5) respectively4
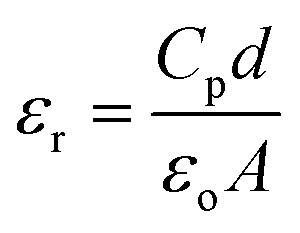
5
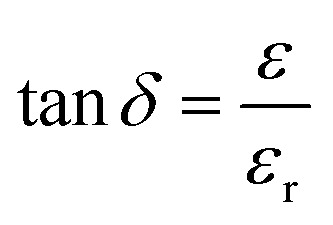
where ‘*C*_p_’ stands for capacitance and *ε* is the imaginary portion of the dielectric function, which is ε* = *ε*_r_ + i*ε*.

### Temperature dependent dielectric properties and Curie–Weiss behavior

**Fig. 8:**
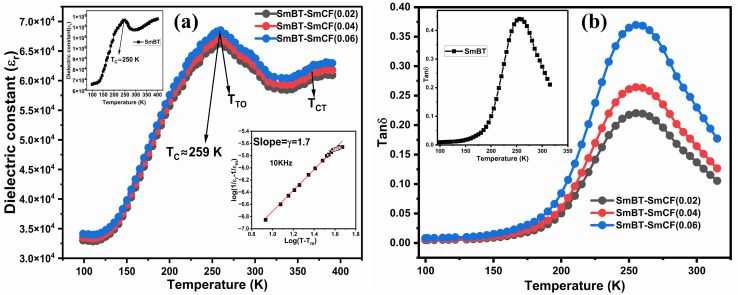
(a) Temperature dependent dielectric constant (*ε*_r_) and insets shows the *ε*_r_ of Ba_0.85_Sm_0.15_TiO_3_ and Curie–Weiss behavior and (b) dielectric loss of (1 − *x*)SmBT–*x*SmCF (*x* = 0.02, 0.04, 0.06).

Fig. 8(a) shows the fluctuation of dielectric constant (*ε*_r_) for particulate composites (1 − *x*)SmBT–*x*SmCF (*x* = 0.0, 0.02, 0.04, 0.06) with temperature (100–400 K) at a frequency of 10 kHz. All of the composites were found to exhibit a high value of *ε*_r_ as a result of the samarium doping,^49^ and their behavior is remarkably comparable to that of previous composites made of BaTiO_3_ and CoFe_2_O_4_.^2,50,51^ Since the process of electron hopping between (Fe^3+^ ↔ Fe^2+^ and Sm^3+^ ↔ Sm^2+^) ions is temperature sensitive, thus an increase in *ε*_r_ with temperature is noticeable. Dielectric polarization rises as a result of the hopping mechanism, which is a temperature-dependent phenomenon. Beyond Curie temperatures, a dramatic decrease in the dielectric constant is seen because the structural transition from the non-centrosymmetric tetragonal phase to the centrosymmetric orthogonal phase cancels the net dipole moment owing to the deformed domain structure.^52^ Comparing composites to published BaTiO_3_,^4^ the Curie temperature (*T*_C_) is lower. The observed reduction in dielectric constant (*ε*_r_) may be ascribed to the dopant ions presence impeding the migration of Ti^4+^ ions in the core area. In composites, when the SmCF phase concentration rises, the internal stress that SmCF grains creates on SmBT grains causes a modest increase in the Curie temperature 250–265 K (*T*_C_) which was also confirmed by *M*–*T* curves. The increase in dielectric constant (*ε*_r_) in composites containing the SmCF phase, that can increase in electron hopping between Fe^3+^ ↔ Fe^2+^ and Sm^3+^ ↔ Sm^2+^ ions. Smaller grains provide a compact and dense structure in the composite material, which facilitates interfacial polarisation and results in higher dielectric constants. Additionally, the reduction in bond and hopping lengths positively corresponds with the increase in electron hopping brought on by the SmCF phase. We analyzed and thoroughly describe the lengths of the octahedral (*B*_oct_) and tetrahedral (*B*_tet_) bonds and are obtained results are given in Table 3. Similar calculations were made for the hopping distances between neighboring ions occupying octahedral and tetrahedral sites.^27^ From SmBT–SmCF(0.02) to SmBT–SmCF(0.06), an increase in Curie temperature is seen in the composites (Table 3). Larger grain size and constrained ferroelectric domains are responsible for this rise in internal tensions. The Curie temperature of SmBT was considerably influenced by Sm^3+^ among all the replaced rare earth ions. The change of *T*_C_ has been shown to be larger for composites. Compared to their lower concentrations, rare earth ion concentrations had a substantial impact on Curie temperature.^53^ Since there is more electron interaction between the octahedral and tetrahedral sites when the hopping duration is shorter, polarisation is thus increased. The following relations were used to compute the hopping lengths between two ions occupying tetrahedral sites and non-tetrahedral sites, indicated by *H*_T_ and *H*_O_:6
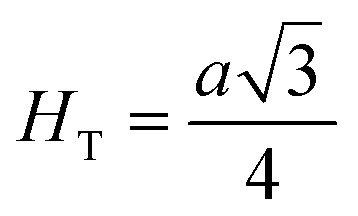
7
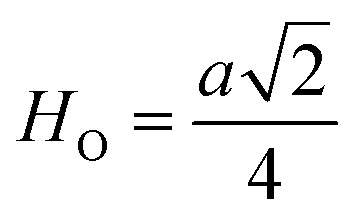
where *a* = 8.34 Å denotes the magnetic SmCF phase lattice constant. Additionally, the following formulae^54^ were used to compute the tetrahedral *B*_T_ and octahedral *B*_O_ bond lengths, abbreviated as *B*_T_ and *B*_O_, respectively.8
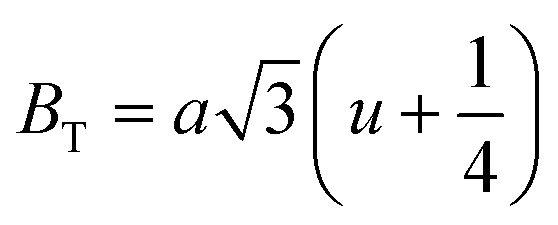
9
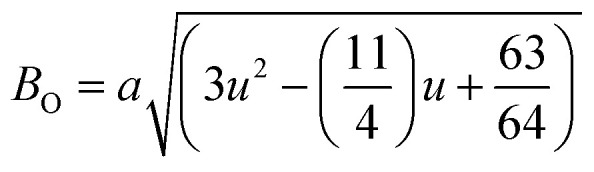
where *u* (*u* = 0.381 Å) is the oxygen positional parameter.

**Table 3:** Various parameters dielectric constant, dielectric loss, Curie temperatures and coupling coefficients of composites sample (1 − *x*)SmBT–*x*SmCF (*x* = 0.02, 0.04, 0.06)

Conc. *X*	*ε*_r(TO)_	*ε*_r(CT)_	*T*_C(TO)_	*T*_C(CT)_	*γ*	tan *δ*	*E*_a_ (eV)	*H*_DC(max)_	*α*_(max)_
0.00	126 116	117 470	250 K	344 K	1.63	0.41	0.57	—	—
0.02	60 061	56 300	259 K	363 K	1.70	0.22	0.55	0.63	1.31
0.04	61 504	57 229	261 K	367 K	1.75	0.26	0.54	0.88	2.14
0.06	62 673	58 506	265 K	368 K	1.79	0.37	0.52	1.13	3.82

**Fig. 9:**
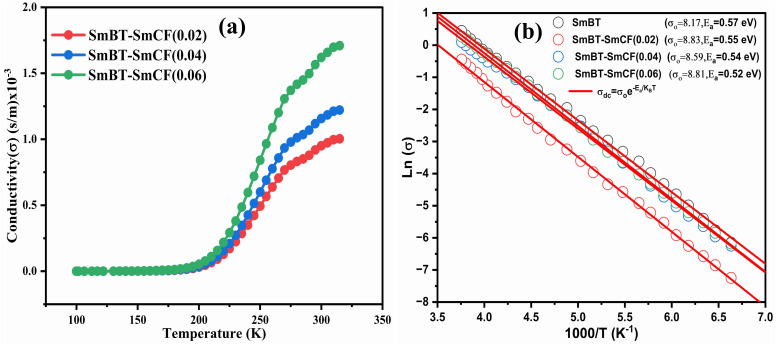
(a) Variation of conductivity with temperature and (b) variation of log(*σ*) with inverse of temperature and shows linear fit of (1 − *x*)Ba_0.5_Sm_0.5_TiO_3_–*x*Co_0.5_Sm_0.5_Fe_2_O_3_ (*x* = 0.02, 0.04, 0.06).

The dielectric data was fitted to the modified version of Curie–Weiss law provided (eqn (10)) for determining the nature of the ferroelectric phase transitions from tetragonal to orthorhombic and cubic to tetrahedral phase.10
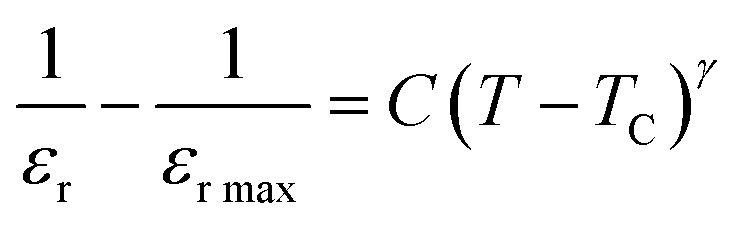
*γ* the parameter determines how the phase transition behaves close to the Curie point, and *C* is a constant. *γ* = 1 and (1 < *γ* ≤ 2), respectively, depict abrupt and diffused phase transitions in the paraelectric zone. In our example, the inset of Fig. 9 displays the fluctuation of ln(1/*ε*_r_ − 1/*ε*_r max_) *vs.* ln(*T* − *T*_C_) of (1 − *x*)Ba_0.85_Sm_0.15_TiO_3_–*x*Co_0.85_Sm_0.15_Fe_2_O_3_ (*x* = 0.02) and rest of them shows same behavior, the slope of which provides the value of *γ*. The obtained values of are all between 1 and 2, indicating that the variable is dispersed.^55^

### Variation of dielectric loss with temperature

Fig. 8(b) shows how the dielectric loss, tan *δ* varies with temperature (100–400 K) at a chosen frequency of 10 kHz for particulate composites (1 − *x*)SmBT–*x*SmCF (*x* = 0.0, 0.02, 0.04, 0.06). According to prior findings,^56^ a rise in tan *δ* is seen as the ferrite content increases, which may be due to the ferrite phase's conducting properties. The absence of domain walls causes tan *δ* to peak close to Curie temperatures, while above these temperatures, tan *δ* shows decrease. When magnetic SmCF phase and ferroelectric SmBT phase are combined, dielectric and ferroelectric characteristics are diluted, which causes the broadness of peaks at Curie temperatures in composite materials. However, the tan *δ* in the SmBT phase is very low, particularly around room temperature, making it a good candidate to construct composites with the SmCF phase. The drop in tan *δ* in composites is caused by the reduction in carrier concentration and non-conducting behavior of grain boundaries of the SmBT phase, which makes particulate composites suitable candidates for ME devices.

### Conductivity formalism

The SmCF phase exhibits a significant conductivity compared to the ferroelectric SmBT phase, which is an insulator with little conductivity. We looked at how conductivity changed with temperature to consider the conductivity of the SmCF phase in multiferroic composites and see how it affected the ferroelectric and magnetic characteristics. The activation energies are investigated over the whole temperature range using the temperature-dependent conductivity investigations. The behavior of conductivity at low temperatures is explained by the Motts law. We calculated AC conductivity (*σ*_ac_) using the dielectric data (*ε*_r_ and tan *δ*) and frequency (*f*) of the applied electric field in relation (11).

11 *σ*_ac_ = 2π*fε*_o_*ε*_r_ tan *δ*

where *ε*_o_ = 8.85 × 10^−12^ F m^−1^, the absolute permittivity.

Plots of *σ*_ac_*vs.* temperature are shown in Fig. 9(a). Due to limited-range polarons, the *σ*_ac_ is modest and almost constant up to certain temperature.^55^ These narrow range polarons are created by the lattice's short-range forces, and the polarisation they induce is only as large as the unit cell. A sudden increase is seen in the conductivity charts above that particular temperature because band conduction dominates polaron-driven conduction.^57^ The conducting SmCF ferrite phase, which is entangled in the ferroelectric SmBT matrix, is the component that gives multiferroic composites their conductivity. An established Verwey–de Bohr mechanism^58^ explains how such particle composites might conduct electricity. According to this process, the conductivity develops as a result of electron exchange (Fe^2+^ − e^−^ ↔ Fe^3+^) and hole hopping (Sm^3+^ − e^−^ ↔ Sm^2+^, Ba^3+^ − e^−^ ↔ Ba^2+^) between the ions with various vacancies.^59^

Temperature has an impact on the electrical conductivity, showing a slight correlation in the range of 100–200 K. Nevertheless, it shows a stronger connection with temperature above 200 K. When subjected to higher temperatures, the conductivity demonstrates exponential behaviour, which can be explained using the eqn (12).12
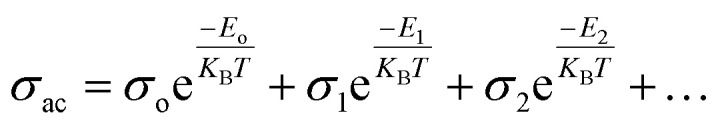


There are certain unspecified constants, namely *σ*_0_, *σ*_1_, and *σ*_2_, and we also have the Boltzmann constant denoted as *K*_B_. The energy labelled as *E*_o_ is linked to intrinsic conduction, while the additional energy terms (*E*_1_, *E*_2_, *etc.*) aid in the phenomenon of hopping conduction. A plot was used to determine the activation energy of composites. The plot involved the logarithm of the conductivity against the reciprocal of the absolute temperature for the SmBT ferroelectric phase and its composites (1 − *x*)Ba_0.85_Sm_0.15_TiO_3_–*x*Co_0.85_Sm_0.15_Fe_2_O_3_ (*x* = 0.02, 0.04, 0.06). The values obtained are shown in Fig. 9(b) and listed in Table 3. There is a notable range of activation energies observed in the temperature range of 285–140 K, with a mix of high and low values. By utilising the small-polaron theory,^60^ we can gain a better understanding of the decrease in activation energy at lower temperatures. In low temperatures, the primary mode of conduction is the electronic process occurring within a narrow energy band. This allows for the transmission of electrical charges through specific states, which is widely recognised as the hopping conduction mechanism.

### Magnetoelectric coupling

In this study, we investigated the relationship between the magnetic SmCF phase and the ferroelectric SmBT phase, focusing on their magnetoelectric connection. We employed the dynamic technique, involving the application of both an AC magnetic field and a static DC magnetic field. The alternating current magnetic field had a frequency of 1 kHz and a consistent amplitude of 1 Oe, while the direct current magnetic field varied in field intensity from 0 to 4 kOe. In order to start the experiment, we electrically polarized every composite, creating a finite electric polarisation. After that, the electrically polarized composites were arranged parallel to the magnetic field's axis. This experimental set-up was used to ascertain the degree of interaction between the ferroelectric SmBT and magnetic SmCF phases. Changes in the ferroelectric characteristics were caused by the interaction of the magnetic field-induced strain in the magnetic phase (due to magnetostriction) and the ferroelectric phase. We were able to assess this magnetoelectric coupling effect's size using the dynamic technique.^53^ By harnessing the magnetoelectric effect, a voltage (δ*V*) can be generated in the material being examined when a magnetic field is applied. The voltage generated was subsequently measured and detected using a lock-in amplifier. The ME coefficient (*α*) was calculated using eqn (13).^61^13
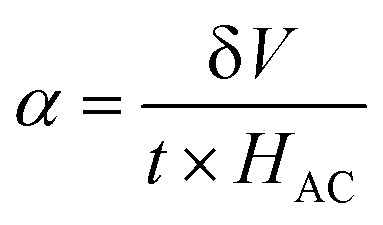
where *t* is the particulate composites thickness, measured in terms of pellet thickness. And δ*V* is given by δ*V* = *V*_(HDC=*H*)_ + *V*_(HDC=0)_.

**Fig. 10:**
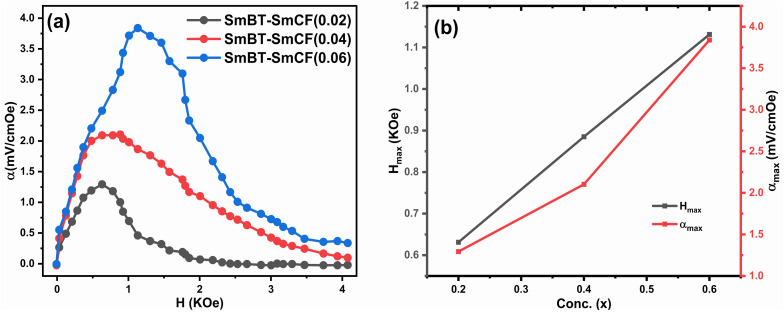
(a) The coupling coefficient (*α*) as a function of the magnetic field (*H*) for composites (1 − *x*)SmBT–*x*SmCF (*x* = 0.02, 0.04, 0.06) (b) shows maximum values (*α*_max_) and certain magnetic field (*H*_max_) for certain ferrite concentration.

According to prior study, the magneto-strictive strain (*λ*) is reliant on the piezo-magnetic coefficient (*q*), which in turn affects the coupling coefficient (*α*).^64^ Mathematically, the coefficient (*q*) is defined as the derivative of *λ* with respect to the applied magnetic field (*q* = d*λ*/d*H*). In the present investigation, a rise in the magnetic field is accompanied by a rise in the magneto-strictive strain (*λ*). For a certain value of the applied magnetic field, a maximum is seen. Beyond this maximum an *α* is shown to go down, and at high magnetic fields, a little change is seen. The decline in *α* is attributed to the magnetic field is applied parallel to the polarisation, supports the observed behavior of *α*. The coupling coefficients are, however, quite small when the *P* and *H* are perpendicular to one another.^62^Fig. 10(a) shows the connection between *α*_max_ and *H* and the Fig. 10(b) shows the highest values (*α*_max_) and certain fields *H*_max_ with respect to ferrite concentration. The primary working principle behind this technique is the creation of strain in the ferromagnetic SmCF phase as a result of the applied magnetic field. After that, by the motion of domain walls, this strain is transmitted to the ferroelectric SmBT phase. The SmBT phase results in the generation of a generated magnetoelectric (ME) voltage. The rise in *α* can be attributed to the heightened elastic interaction between the SmBT and SmCF phases.^63^ In a given field, all composites of the coupling coefficient show a peak, which is then followed by partial saturation. Based on the *M*–*H* hysteresis loops, it becomes evident that the peak is situated close to the saturation field of 1.5 kOe. The highest values are generated at the saturation field, when the maximum strain is transferred from the magnetic SmCF phase to the ferroelectric SmBT phase.^64^ Given the larger content of the magnetic phase SmCF, it is not surprising that SmBT–*x*SmCF (*x* = 0.06) has a maximum value of *α*, followed by 0.04 and 0.02. Because 0.06 has larger grains and high porosity, maximal strain transmission across grain boundaries is possible, and this results in the higher value. This study's detection of a high coupling value (Table 3) in comparison to previous rare-earth doped particle composites^43,46,50,65,66^ is an important finding. The incorporation of samarium into the barium titanate lattice leads to an improved SmBT phase and higher polarisation dielectric constant, which is linked to the increase in *α*. Overall, the samarium doped particulate composites show promise for magnetoelectric devices due to the enhanced coupling coefficients resulting from the notable polarisation of the SmBT phase.

## Conclusion

In summary, our study was centered on examining the characteristics of multiferroic composites derived from the (1 − *x*)Ba_0.85_Sm_0.15_TiO_3_–*x*Co_0.85_Sm_0.15_Fe_2_O_3_ framework, using 0–3 particulate composite scheme. Rietveld refinement of the samples revealed a tetragonal structure in Ba_0.85_Sm_0.15_TiO_3_ (SmBT) and a cubic spinel secondary phase in Co_0.85_Sm_0.15_Fe_2_O_4_ (SmCF). The saturation magnetization and magnetic moments were found to increase with an increase in the concentration of the magnetic phase (SmCF). The results of domain analysis revealed a complex multi-domain architecture, which was attributed to the pinning phenomenon induced by the ferroelectric SmBT phase. The observed correlation between the concentration of the non-magnetic SmBT phase and the Curie temperature (*T*_C_) suggests a reduction in magnetic linkages. The dielectric studies revealed the introduction of the SmCF phase resulted in an elevation of both the Curie temperature (*T*_C_), dielectric constant (*ε*_r_) and dielectric loss (tan *δ*) as a consequence of the internal stresses and electron hopping phenomena. Based on the results of the conductivity analysis, it can be inferred that the SmCF phase played a significant role in enhancing the conductivity of the composites. The composite samples show significant improvements in all ferroelectric characteristics. This, in turn, led to an improved coupling between the ferroelectric and magnetic phases. Magnetoelectric coupling studies shows the samarium doped particulate composites are good candidates for magnetoelectric devices owing to the improved coupling coefficients caused by significant polarisation of the SmBT phase. The observed phenomenon of an elevated magnetoelectric coupling constant signifies an increased degree of interaction between magnetic and electric fields. This enhanced interaction leads to a more proficient conversion of electrical energy into its magnetic counterpart, thereby indicating an increase in overall energy efficiency.

## Data availability

All data generated or analyzed during this study are included in this article.

## Author contributions

Showket Ahmad Bhat: conceptualization, methodology, experimentation, validation, formal analysis and writing original draft. Prof. M. Ikram: conceptualization, resource, visualization, validation, supervision, and revision.

## Conflicts of interest

The authors state that they have no financial conflicts of interest or personal ties that could have influenced the research presented in this study.

## Supplementary Material

RA-014-D4RA01360C-s001
