# Supplementary material for: Synergistic magnetoelectric enhancement in 0–3 particulate multiferroic composites: unveiling the exceptional interplay of Ba0.85Sm0.15TiO3 and Co0.85Sm0.15Fe2O4 phases for superior energy conversion
Source: RSC Adv. 2024 May 17;14(23):15915–28. doi: 10.1039/d4ra01360c (PMC11099903; doi:10.1039/d4ra01360c)
Supplement: RA-014-D4RA01360C-s001 [file RA-014-D4RA01360C-s001.pdf]

Electronic Supplementary Material (ESI) for RSC Advances.
This journal is © The Royal Society of Chemistry 2024

Supporting Data

EDS data

Table 1: *Weight percentage of elements of composites (1-x)SmBT-xSmCF (x = 0.00,1.0,0.02,0.04, 0.06).*

Element	*Weight % SmBT*	*Weight % SmCF*	*Weight % SmBT-SmCF (0.02)*	*Weight % SmBT-SmCF (0.04)*	*Weight % SmBT-SmCF (0.06)*
O K	*20.41*	*25.74*	*20.52*	*20.62*	*20.73*
Ti K	*20.35*	*-*	*19.94*	*19.54*	*19.13*
Sm K	*9.5*	*9.07*	*9.49*	*9.48*	*19.47*
Fe K	*-*	*45.04*	*0.90*	*1.80*	*2.7*
Co K	*-*	*20.15*	*0.40*	*0.81*	*1.21*
Ba L	*49.64*	*-*	*48.65*	*47.65*	*46.66*

Raman Spectra

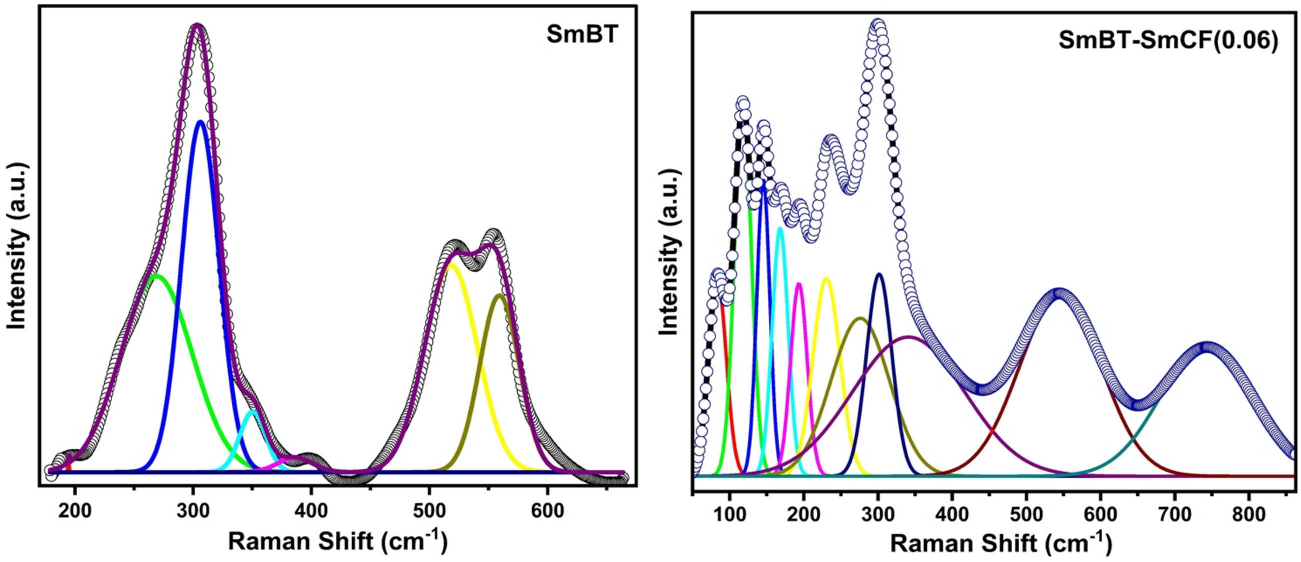

Figure 1*:Deconvoluted Raman spectra of composite (1-x)SmBT-xSmCF (x=0.00,0.06).*

X-ray photoelectron spectroscopy

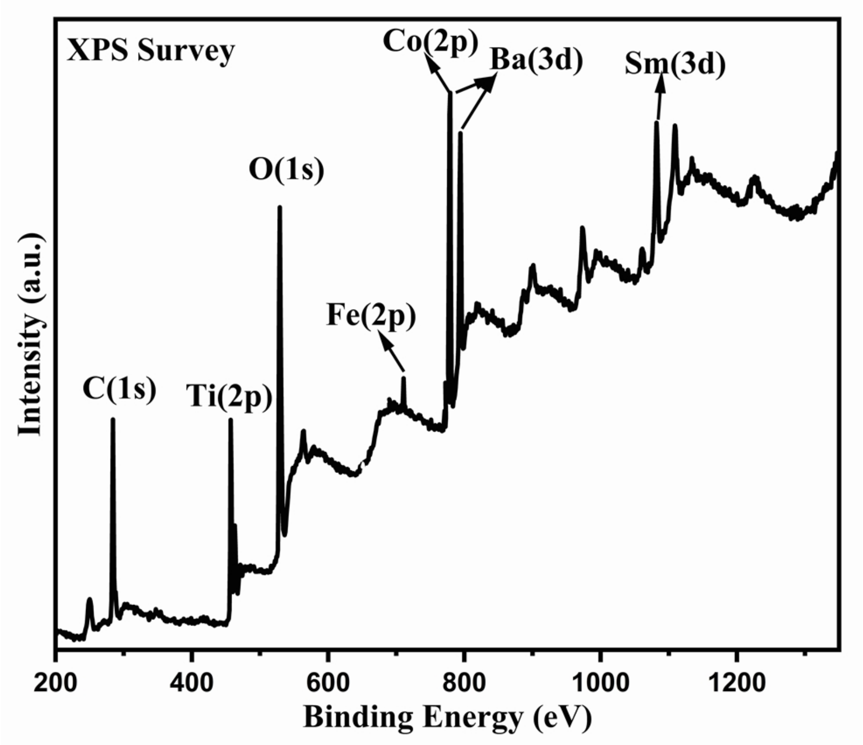

Figure 2: *XPS survey spectrum of (1-x)SmBT-xSmCF.*

Table 2: *XPS peak positions and FWHM values of (1-x)SmBT-xSmCF .*

Element Name	Levels	**Fitted peak position (eV)**	**FWHM (eV)**
Ba 3d	$3d_{5/2}$, $3d_{3/2}$	794.81,779.45	3.24
Ti 2p	$2p_{3/2}$, $2p_{1/2}$	457.9,463.71	2.45
Sm 3d	$3d_{5/2}$, $3d_{3/2}$	1083.08,1109.43	2.13
Co 2p	$2p_{3/2}$, $2p_{1/2}$	779.7,796.4	3.20
Fe 2p	$2p_{3/2}$, $2p_{1/2}$	710.5,733.4,724.6	3.31
O 1s	1s	529.38,532.24	1.20
